# Schumpeter’s picture of economic and political institutions in the light of a cognitive approach to human behavior

**DOI:** 10.1007/s00191-015-0421-9

**Published:** 2015-09-04

**Authors:** Massimo Egidi

**Affiliations:** LUISS Guido Carli, Viale Pola 12, 00198 Rome, Italy

**Keywords:** Cognition, Bounded rationality, Democracy, Information and knowledge, Communication, Persuasion, B31, D820, D830

## Abstract

Schumpeter’s theory of democracy can be read through the lens of the cognitive approach to rationality. Schumpeter himself constructed his theory on the basis of his (neglected) conception of *conscious rationality*, which considers the process of thinking as composed of conscious/deliberate and unconscious/automatic components. The prevalence of the deliberate over the automatic component can occur in different degrees; as a consequence, individuals exhibit different levels of conscious rationality. Schumpeter makes clear that an essential attribute of democracy is its being a system of government capable of working notwithstanding a low degree of conscious rationality among its citizens. Given this condition, the process of political communication and persuasion can lead to two very different outcomes: a fair social construction of the democratic institutions, in which the struggle for the vote is achieved through a critical debate among leaders and citizens; and an unfair construction, based on the prevalence of emotive forces of persuasion over rationality and on cheating of the leaders at the expense of their citizens. Schumpeter suggests that the main element that fosters a fair construction is the effectiveness of competition, which can advance the rational elements in the political debate and the self-determination of the citizens’ will: a slow process that – he warns - may be effective only in the long run, and does not preserve democracy from the risk of decline.

## Introduction

Part IV of Schumpeter’s *Capitalism*, *Socialism and Democracy* is characterized by a brilliant and realistic picture of capitalism supported by new and powerful theoretical ideas, which in my opinion have not thus far fully realized their potential. If we consider the pages dedicated to entrepreneurship and to democracy, we see that the intellectual toolbox he used in his previous works - based on the economic thought of the French and Austrian schools, and to some extent limited by the implicit contrasts between those two schools - has undergone a radical evolution.

Schumpeter’s new ideas on the nature of democracy also entail new ideas on the nature of human behavior within institutions, and these ideas conflict to a certain extent with the *corpus* of traditional economic doctrines. It follows that there are only two alternatives: either to attempt to explore whether its depiction of market and democracy can be interpreted within a unified frame, or to consider the analysis of market and of democracy as separate conceptual domains. The latter is the traditional approach, while the former – I suggest - implies a reading of Schumpeter’s thought in the light of the recent advancements in the cognitive approach to human behavior: a mental experiment that I propose in the following pages.

The chances of successfully applying this approach are facilitated by the fact that Schumpeter himself reflected on the meaning of rationality in many parts of his works. He started from the classical definitions provided by Max Weber and then moved significant steps ahead, urged by the necessity to encompass under the same notion of rationality the different kinds of contrasting behavior manifested by the subjects that populate his theory : entrepreneurs, consumers, citizens and political leaders are in fact depicted as carriers of different kinds and levels of rationality. In what follows, I will show that Schumpeter’s theory is, to some extent, a forerunner of the basic principles of bounded rationality. In this introduction, I will summarize Schumpeter’s main assumptions on rationality and their consequence to the architecture of his theory of democracy, by extracting Schumpeter’s’ ideas and interpreting them through the concepts of cognitive psychology.

1. Schumpeter’s analysis presupposes limits on the rationality and knowledge of individuals both in economics and politics. These limits are clarified and explored under the label of “conscious rationality”: in his view individuals may have *different degrees of conscious rationality*, and so, to practice rationality in a given domain, it is necessary to hold specific competence and knowledge. When competence is very high, as is the case of entrepreneurial activity, rationality can be described as a process of *creative response*, i.e., *of discovery under uncertain conditions*, analogous to Simon’s procedural rationality. When competence is missing or is very modest, the emotional and routine elements of reasoning supplant logical thought and individuals are exposed to irrational external messages, forms of persuasion and advertising, both in the economic and political arena. (Schumpeter (2003), 363)
2. Given that an economic or a political decision requires expertise, individuals may decide with full competence and rationality only in a limited number of domains. The element that reinforces competence is the familiarity with and proximity to a given decision context, and, for this reason, at least a part of economic decisions can be made in a more competent way than political ones. To paraphrase Schumpeter, when we move away from the private concerns of the family and the business office into those regions of national and international affairs that lack a direct link with the private concerns, individual volition ceases to fulfill the requirements of conscious rationality : the degree of conscious rationality dumbs down to a very modest level. (Schumpeter (2003), 259–261)
3. Democracy is based primarily on delegation because of the limits of competence and knowledge of its citizens. Leaders are supposed to possess the specific competence in politics that is modest or missing among those they represent. They collect the will and reflect the opinions of their constituency, but to a limited extent; in fact, as with the entrepreneur, the political leader may be an innovator, able to bring forth new ideas and proposals that may conflict with the common feeling or the average opinion of his constituency. Such an ability requires vision and the *ability to persuade*. This means that leaders “may fashion and, within very wide limits, even create the will of the people. “(Schumpeter (2003), 263)
4. Of course, a leader may use his persuasive power in two ways: in a *positive* sense, if he tries to convince his constituency by virtue of an attempt clearly and transparently to expound his proposals; or in a *negative* sense, if he conveys information, ideas or ideologies to the citizens in a systematically distorted way. In the latter case, the leader does not appeal to rationality but rather uses communicative and persuasive techniques typical of advertising, based on psychological associations and related emotions. (Schumpeter (2003), 263)
5. The previous points imply that, in a democracy, leaders can manipulate opinions and produce systematic distortions of the popular will: this is a crucial aspect of Schumpeter’s analysis. It follows that if we do not want to relegate it to a pure description due to his acute insight, it is necessary to appeal to the modern theory of psychology of cognition, in which the characteristics of reasoning and, in particular, the role of persuasion and manipulation, has been extensively studied and experimentally proved. To this end, the next section is dedicated to the connections between Schumpeter’s notion of *conscious rationality* and the modern theory of bounded rationality.
6. Schumpeter depicts democracy as an institution that allows people to achieve political decisions *without necessarily having a high degree of awareness and competence*. The main problem that Schumpeter has to solve in sketching the fundamentals of democracy is conceiving institutions that produce rational, or at least reasonable, public decisions *despite* substantial limits in terms of individuals’ rationality and competence. He highlights the observation that a defining property of democracy is its being an institution that may function even if the degree of awareness, competence and analytical effort of citizens is low, provided that it is balanced by responsible behavior on the part of its leaders.
7. Naturally, this view attributes fragility to democratic institutions, insofar as they are exposed to serious risks: risks that emerge if the design of the internal workings of democratic institutions does not allow the process of collective decision-making to work properly with a transparent allocation of responsibilities. In this case, ambitious leaders may implement various forms of shirking that can lead to a dangerous undermining of the quality of democracy. This is the reason why *competition* is an essential element of democracy: it is the only method that permits the emergence of a definite will among citizens, by raising the level of conscious rationality through a pacific political struggle. The only method that reduces the citizens’ chances of being fooled by irresponsible leaders and assures a good quality of democracy is a process of reducing the citizens’ competence gap, which, according to Schumpeter, can only occur in the long run.

## Conscious rationality and bounded rationality

Schumpeter’s ideas on rationality were advanced on occasion in many of his major publications, but it is in “Rationality in Social Sciences”, a document written when Schumpeter was beginning to work on his *Capitalism*, *Socialism and Democracy*, that his views are most carefully expounded. 1 Here he advances the idea of *conscious rationality* by considering the process of thinking as composed of conscious/deliberate and unconscious/automatic components. The prevalence of the deliberate component over the automatic one can happen to different degrees, and, in consequence, individuals may exhibit different degrees of rationality. This element implies that the analyst, while applying a rational schema to the actor’s behavior, must take into account that “models of rational action do not necessarily imply conscious rationality on the part of the actors” (Schumpeter 1947, 584).

Thus, if we take this concept seriously, we soon understand that Schumpeter uses it to explain why the same individual can be fully rational in his economic activity and scarcely rational when making political decisions. The key aspect is that an individual may have different degrees of conscious rationality depending upon the domain of competence in which he operates: a low level of conscious rationality characterizes a citizen’s voting decision if he does not (and sometimes does not want to) acquire sufficient expertise to make highly competent decisions in the field of politics. The same person may exhibit a high degree of conscious rationality in a different domain, for example, economics.

A similar distinction can be found in the domain of economics, where a consumer’s behavior may be characterized by a modest degree of conscious rationality, while an entrepreneur’s behavior may unfold at the highest degree of rationality.

In *Rationality in Social Sciences* 2 Schumpeter explicitly declares that the assumption of conscious rationality is an essential pillar for the microfoundations of his theory of democracy: his new, “reformed” theory of democracy considerably drops the hypothesis that all individuals make their choices with conscious rationality at the highest level.

As I have said, Schumpeter must resort to a view of rationality that is broader and richer in respect to the one codified by Lionel Robbins’s in his *Nature and Significance of Economic Science* (1932), to encompass the different types of behavior exhibited by the actors who populate his depiction of democracy and capitalism.

I will start by examining the notion of conscious rationality within the domain of the *Theory of Economic Development*, and subsequently (next paragraph) I will discuss the domain of the theory of democracy.

The dualism between a stationary regime (circular flow) and an evolutionary regime (development), on which the *Theory of Economic Development* was constructed, naturally required a description of two different ways of making economic decisions: the routinized behavior that characterizes the circular flow and the innovative behavior that describes entrepreneurial action.

Richard Langlois (1996) clearly underlines the argument:

“The circular flow is a closed world. There may be changes in data and various shocks to which the agents must adapt, but there is nothing fundamentally new. It is the entrepreneur whose task it is to introduce novelty. And novelty, for Schumpeter, brings with it problems not found in the circular flow. The entrepreneur who steps outside the confines of the ordinary

“…must really to some extent do what tradition does for him in everyday life, viz. consciously plan his conduct in every particular. There will be *much more conscious rationality* in this than in customary action, which as such does not need to be reflected on at all; but this plan must necessarily be open not only to errors greater in degree, but also to other kinds of errors than those occurring in customary action.

.... How different a thing this is becomes clearer if one bears in mind the impossibility of surveying exhaustively all the effects and counter-effects of the projected enterprise. Even as many of them as could in theory be ascertained if one had unlimited time and means must practically remain in the dark. … Here the success of everything depends upon intuition, the capacity of seeing things in a way which afterwards proves to be true, even though it cannot be established at the moment, and of grasping the essential fact, discarding the unessential, even though one can give no account of the principles by which this is done” (Schumpeter (1934) 85).

Langlois comments

By stepping outside the bounds of routine, the entrepreneur extends the sphere in which conscious planning is necessary for successful action. But, because of “bounded rationality,” he or she cannot in fact act rationally in the strict sense and, as a result, must rely on intuition. Thus entrepreneurship à la Schumpeter is an extra-rational activity from the point of view of the logical conception of rationality — though it is very much a rational activity according to Schumpeter’s own meaning of the term. (Langlois, (1996) 5)

This comment helps us to consider entrepreneurial activity as a “*creative response*” fundamentally linked to a process of *discovery* under uncertain conditions, a process analyzed in great depth by Herbert Simon in his studies on procedural rationality. 3A good sample of this activity can be viewed when we examine the behavior of a chess master: nobody would consider the chess master to be irrational when he is playing against his opponent, simply because his behavior cannot be reduced to the standard model of optimization. The point is that, in this context, rationality (in a broad, Weberian sense) is no longer a process of optimization: The chess playing activity cannot be reduced to optimization because the winning strategy, which already exists, is not *practically* computable. 4 Rationality can thus be interpreted as optimization only under the condition whereby the available alternatives (deterministic or stochastic) are known in advance. In conditions of procedural uncertainty, not all alternatives can be discovered and the *rational* strategy for a player is to discover more strategies than his opponent.

This kind of process has been extensively studied by Herbert Simon and many other scholars over the past 40 years, considering chess as an ideal context 5 for the empirical study of expert decision making. The players’ mental mechanism, which can be defined as “procedurally rational”, is described by Simon in the following way:

“Research in cognitive psychology in recent years has made great progress in understanding human expertise […] First, expertise is based on extensive knowledge […] A world-class expert in any field (several domains have been studied in some detail) holds in memory some 50,000 chunks (familiar units) of relevant information. This body of knowledge is stored in the form of an indexed encyclopedia […]. Associated with each chunk is a set of cues which, whenever evoked by a stimulus, will provide access to that chunk in semantic memory. […]

Armed with knowledge stored in his [memory], the expert is prepared (but only in the domain of expertise) to respond to many situations “intuitively”—that is, by recognizing the situation and evoking an appropriate response—and also to draw on the stored [knowledge] for more protracted and systematic analysis of difficult problems.” (Simon (1991) 129)

The mental mechanism is the same for all players, but works at different levels, depending upon the degree of expertise of the player (from novice to great master). The “ability to see things in a way which afterwards proves to be true”, the intuition of a great master is, in reality, a complex searching activity guided by *heuristics* in which the master “sees” the evolution of the match a number of steps further than his opponent.

We could interpret Schumpeter’s description of the capacity for innovative activity or “seeing things in a way which afterwards proves to be true, even though it cannot be established at the moment”, with an immediate application of the theory of bounded rationality. With this goal, imagine a club of people who decide, during their vacation, to hold a chess tournament. Suppose all of them in the past have been trained at an intermediate level, and have approximately the same (modest) degree of competence. This means that they know the most common basic strategies, and therefore each of them understands the strategy of his opponent after the opening moves and can reasonably try to predict the next move. Now, a grand master lands in the club: every player in the club will be unable to predict the grand master’s moves because he draws upon a vastly wider repertoire of strategies and can explore in much greater depth than his opponent the possible sequences of moves-countermoves at his disposal. To the eyes of the club players, the master’s strategy is *unpredictable*, and exhibits precisely the characteristics that Schumpeter attributes to an innovator:

“From the standpoint of the observer who is in full possession of all relevant facts, it can always be understood *ex post*; but it can practically never be understood *ex ante*; that is to say, it cannot be predicted by applying the ordinary rules of inference from the pre-existing facts.” (Schumpeter (1947), 150)

So far, then, we could explain innovation purely on the basis of the *gap of competence* 6 between the innovator and the other individuals.

By virtue of a vast empirical research, Simon created computer programs to simulate the search process, and the attempt proved extremely successful: an heir of Simon’s programs, Deep Blue, won against a world champion on February 10, 1996, when it defeated Garry Kasparov. We could thus say that a program may display an *artificial competence* superior to the human one, and, once again, the competence gap is the right criterion that fits with Schumpeter’s requisites for creativity.

Despite the success, programs like Deep Blue exhibit significant limits: they can brilliantly reproduce the heuristic search, and also simulate *adaptive changes* of strategy in a defined context; but to this day a program that simulates and reproduces the creative activity for *every* problem solving context, and especially for scientific discovery, has not been created. This does not destroy our criterion but instead renders it more general: a *creative change* requires a competence that is still not available on the basis of existing knowledge. This definition is coherent with the two key features of creative action advanced by Schumpeter, i.e., unpredictability and the impossibility to be understood *ex ante*.^7^ Moreover, it implies that creativity may indeed have a component that is due to chance, but is nonetheless robustly rooted in human competence and intelligence.

In sum, what we have is a modern and experimentally validated theory of procedural rationality that explains the features of what Schumpeter intuitively calls “conscious rationality at a high degree”. In conditions of *procedural uncertainty*, 8 when an individual is highly competent in a particular domain, a creative decision is the outcome of a complex process of research. I maintain that this activity is highly rational.

## Conscious rationality and dual model of reasoning

The theory of bounded rationality can also be applied in order to understand behavior in conditions of low competence, given that, according to Schumpeter, it happens for the consumer’s choice and the citizen’s political choice. To understand better this case, we have to explore further developments of the theory.

As we have seen, Simon’s theory maintains that, in the course of acquiring their skill, chessplayers store chunks in long-term memory corresponding to *patterns* of pieces. The recall from long term memory, during the match, is fast and automatic, and constitutes the basis for the conscious process of symbolic manipulation of the mental items that have been recalled. Beyond Simon’s findings, this dualism between *unconscious* and *deliberate* aspects of the process of thinking has been further explored over the past years, also outside of the context of chess, as an early experiment by Schneider and Shiffrin (1977) shows.

Thinking is again considered to emerge as an interaction between two kinds of processes, one of which is automatic, and allows the retrieval of items from long term memory: it proceeds without the subject’s control and without necessarily demanding attention. It is called “automatic” or “intuitive”. The second process is called “controlled” or “deliberate” and is the conscious activation of thoughts that require attention, is capacity-limited, serial, and controlled by the subject. This approach is called the “dual model account of reasoning” 9

In his theory of “conscious rationality”, Schumpeter is aware of the distinction between automatic/unconscious and explicit/conscious components of the thought, and preempts the basic distinction of the dual model in a remarkable way:

“I suspect that part of the opposition my theory of subjective rationality met in our group, especially from Professor Parsons, is due to my infelicitous terminology. Perhaps [….] I should not have used the word “conscious” since automatization of often repeated actions will make forms of behavior subconscious which are, nevertheless, included in my conscious rationality: if a mathematician solves a differential equation in the best known manner he is being “consciously rational” in my sense even if, in a particularly simple case, he writes down the solution quite mechanically.” (Schumpeter 1947, 586).

This distinction is evident in many parts of *Capitalism Socialism and Democracy*, albeit in a less precise way, in particular in Part III, Human Nature in Politics.^10^

Beyond the dualism to which I have referred, a third element that plays a key role in reasoning is the impact of emotions. This element is stressed by Schumpeter in many parts of his works, especially when he emphasizes the impact of political advertising on citizens.

In the modern approach, it is widely accepted that emotions are a permanent element that influence behavior. I will not seek to summarize the vast literature on this issue, but simply recall the “somatic marker hypothesis” through which Damasio (1994) and Bechara and Damasio (2005) explain how affective reactions ordinarily guide and simplify decision making. 11

One reason to summarize briefly some of the characteristics of the dual approach to thinking is that it allows us to provide a modern background for Schumpeter’s outsight that social rules and norms may seriously curtail the ability of a citizen to make inferences and to decide autonomously.

To this end, the advancements due to Kahneman and Tversky are most helpful: they explore the interferences between the items memorized in long term memory, which come to mind automatically, with the process of conscious deliberation. On the one hand, the deliberate process may modify, correct or reject intuitive thoughts. On the other, the working of the deliberate process may be undermined by the automatic one, which can override with intuitive answers the process of deliberation.

This last process is particularly interesting in explaining Schumpeter’s observation of the behavior of citizens facing a political choice. In the domains in which the competence of an individual is very limited, the semantic content of the thought may override the individual’s logical ability: prior knowledge and in particular social rules and norms, may dumb down the ability to make inferences. This phenomenon is demonstrated by two key experiments in cognitive psychology: the *belief*-*bias* effect, and the *Wason selection task* 12 . The first one seeks to create a conflict between responses based upon a process of logical reasoning and those derived from prior belief about the truth of conclusions. The experiments show a very strong impact of prior knowledge on the reasoning process. 13

The second experiment, the “Wason selection task”, clearly demonstrates the impact of norms on logical reasoning: in particular, it shows that if the task is described as a problem of social regulation, it can either be solved in a very easy way or cannot be easily solved, in relation to the nature of the norm that is implied. (See Appendix). As a consequence, social norms may have a major role either in *fading* or in *making easier* the reasoning process. This fragility of the human capacity for reasoning can be corrected only in the long run because, according to the dual model, the recall of items that could hinder or foster the responses to given problems cannot be consciously controlled: the memorized items have different *degrees of accessibility*. In consequence, the only way to modify accessibility is through a slow process of learning through which the influence of a hidden norm can be marginalized.

According to Kahneman “the acquisition of skills selectively increases the accessibility of useful responses”.^14^ Chess provides a clear example: the more expert the chess player becomes, the more sophisticated – and different – becomes the heuristic that emerges when faced with the same distribution of pieces on a given chess board. Therefore individuals, improving their skill on a particular domain through prolonged practice, may modify and increase accessibility to the memorized items. As we will see later, Schumpeter points to exactly the same problem, i.e., the fact that citizens, however well informed, can achieve more rational and competent political choice only with time and effort, while, in the short run, the heuristic answer to a problem is rapid and out of the full deliberate control and therefore subject to errors and biases.

We thus have a set of controlled experimental data and a basic analytical toolbox for understanding and justifying Schumpeter’s outsights about the behavior in the domain in which an individual does not possesses a high level of knowledge and competence.

According to Schumpeter, it is essential to consider the case in which the individuals are consciously rational to a *modest degree*, which happens for every individual in some area of competence, and is particularly common in the area of political choice.

“The old theory of democracy as formulated in the seventeenth and eighteenth centuries *presupposes* degrees of awareness of one’s interests, clearness of ends, rationality in the perception and use of means and, most important of all, *accessibility* to rational argument which are altogether unrealistic. A reformed theory of democracy could still use, to a considerable extent, rational schemata, but it would have to drop, not wholly but also to a considerable extent, the hypothesis of *conscious rationality*;(Schumpeter (1984), 585) 15

In *Capitalism Socialism and Democracy* the limits of human rationality are more precisely highlighted. Here Schumpeter considers these limits as a universal trait of human thinking, which characterizes both consumer choice on the market and citizen’s voting decision in the political arena.

“Economists, learning to observe their facts more closely, have begun to discover that, even in the most ordinary currents of daily life, their consumers do not quite live up to the idea that the economic textbook used to convey. On the one hand their wants are nothing like as definite and their actions upon those wants nothing like as rational and prompt. On the other hand they are so amenable to the influence of advertising and other methods of persuasion that producers often seem to dictate to them instead of being directed by them.” Schumpeter (2003), 257

The ability of individuals to reflect, to form independent opinions and to decide is even more limited in the domain of political decisions:

“even if there were no political groups trying to influence him, the typical citizen would in political matters tend to yield to extrarational or irrational prejudice and impulse. The weakness of the rational processes he applies to politics and the absence of effective logical control over the results he arrives at would in themselves suffice to account for that.” Schumpeter (2003), 262

In conclusion, Schumpeter’s broad view of rationality contains *in nuce* and in a simplified way certain principles on rationality that are strikingly similar to the ones that preside over Simon’s theory of bounded rationality.

With his conceptual distinction between high and low degree of conscious rationality, he can encompass two different kind of behavior: the leader/innovator and the citizen/consumer. The first form of behavior arises from situations of unpredictability, uncertainty and complexity, and accordingly is treated by Schumpeter as a problem of *search* and *innovation*. His description of innovative activity fits with the notion of “procedural rationality” that describes the search and decision process of an expert in conditions of uncertainty.

As regards the second form of behavior, Schumpeter holds that consumer choice and the political choice by the citizen, given that they are conducted in domains where their competence is low, are characterized by a distinctly low level of “conscious rationality”: in these situations, the limits of rationality are described - in a vivid and rich manner - as dominated by extrarational impulses, in a way that again fits with the dual model account of reasoning, and in particular with the experimental data on the biasing role of social norms on human reasoning.

Thus we have collected the basic elements of modern cognitive psychology that support Schumpeter’s ideas on rationality and innovation, and can further use it to understand the limits of both the market and democracy.

## From deliberative to representative democracy: human nature in politics

With the limits of rationality in mind, let us turn to what Schumpeter calls his “reformed theory of democracy”

“The eighteenth-century philosophy of democracy may be couched in the following definition: the democratic method is that institutional arrangement for arriving at political decisions which realizes the common good by making the people itself decide issues through the election of individuals who are to assemble in order to carry out its will.” Schumpeter (2003), 250

This “classical” conception of democracy is based on three important pillars: the idea that there exists a common good that people can achieve, that this good may be expressed through a shared will (*Volonté Général*), and that the Common Will is achieved via a process of deliberation amongst the population. One of the more important, indeed perhaps the most striking point of departure of Schumpeter from the classical theory of democracy, is his critical analysis of these three concepts.

“There is … no such thing as a uniquely determined common good that all people could agree on or be made to agree on by the force of rational argument. This is due not primarily to the fact that some people may want things other than the common good but to the much more fundamental fact that to different individuals and groups the common good is bound to mean different things.” Schumpeter (2003), 251

Here, the implicit idea is that the existence of a variety of interests, beliefs, values, opinions within a society is a measure of the degree of freedom of its citizens; the main problem is, therefore, which institutional form can guarantee a balance of citizens’ conflicting opinions, values and interests. The process by which political movements lead a (limited) part of the population towards converging opinions and will is in any case highly uncertain, complex and difficult. The question is how an agreement among many individuals can be constructed, how long it can remain stable, and what are the communicative structures that may lead to the achievement of a common will, in conditions of much dispersed knowledge and limits to rationality.

“… our chief troubles about the classical theory centered in the proposition that “the people” hold a definite and rational opinion about every individual question and that they give effect to this opinion—in a democracy—by choosing “representatives” who will see to it that opinion is carried out. “Schumpeter (2003), 269

In reading Schumpeter through the lens of cognitive psychology, we should say that autonomous reasoning requires a high level of cognitive effort, and that the effort is related to the degree of relevance that individuals ascribe to the issues in discussion. It requires attention and motivations; attention and motivations are, in turn, influenced by *proximity* and *familiarity* to the problems. Schumpeter claims that simply because the proximity and familiarity of citizens as regards political choice are very modest, they maintain their cognitive efforts at a low level, and therefore their autonomy of thought is very low, and persists even in the face of any effort to increase their awareness through critical analysis and information.

“[….] thus the typical citizen drops down to a lower level of mental performance as soon as he enters the political field. He argues and analyzes in a way which he would readily recognize as infantile within the sphere of his real interests. He becomes a primitive again. *His thinking becomes associative and affective*.” (Italics added) 16 Schumpeter (2003), 262

This gap of competence, of course, is variable within the population: only a minority of individuals make full and critical use of the available information, but, more importantly, this level is not a “given” for all time. In fact, while overstressing the limits of human mental performances in the face of political decisions, Schumpeter depicts a process in which everyone, under the right conditions, can increase his degree of awareness. When this happens, and an individual decides to increase his participation in politics, his thinking, which is permanently characterized by a complex dynamic between *autonomous* and *induced* individual will, may achieve the highest levels of autonomy and consciousness. The question is to understand what elements trigger or prevent the emergence of a higher participation among citizens. It is a question that has been explored in great depth in recent years both within cognitive sciences and within the disciplines of communication and networking,^17^ as we will see next.

## The dualism between *autonomous* and *induced* individual will

Schumpeter’s picture of the role of persuasion and advertising plays a significant role because it describes limits and risks implicit to the relation between a leader and his constituency. It is based on an important dualism between *autonomous* and *induced* individual will. This dualism has been largely studied in contemporary psychology, and the origin of induced will is related to the dualism between *unconscious* and *deliberate* aspects of the process of thinking.

The experimental evidence of the interferences between the deliberate and the intuitive components of reasoning allow us to identify the conditions under which the unconscious and emotional elements can prevail over the deliberate component; these conditions are vividly depicted in section III of chapter XXI of *Capitalism Socialism and Democracy*, in which Schumpeter warns that, under particular conditions, political behavior can be reduced to “associative and affective” conduct, beyond rational control.

By clarifying the ways in which the deliberate, the unconscious and the emotional components play a role within the process of reasoning, we can understand the ways through which persuasion has a central impact on the process of reasoning and can frame Schumpeter’s analyses of the effects of advertising on individual will.

In common language, the concept of “persuasion” has a broad semantic scope, meaning the art of modifying the ideas or behavior of the other. Social psychology has developed an extensive degree of literature on this topic, often in relation to the study of advertising.

It is widely acknowledged that, under some conditions, persuasion may work as a process through which one subject modifies another’s ideas, where both parts are acutely conscious of the ongoing transformation, as occurs in teacher-student relations; conversely, under other conditions, persuasion flows through processes that modify the ideas of the other in a way that is, at least partially or totally, out of the conscious control of the recipient, as occurs in advertising.

The former kind of process requires a high degree of mental elaboration by the recipient, and so it may be reinforced through debate and critical discussion; the latter flows through unilateral communication and pre-selected information, and is received by the recipient without highly active elaboration.

The dualism between autonomous and induced will can be explained within the dual model account of reasoning because the impact of the process of persuasion on an individual may be remarkably different depending upon the degree of his mental active reaction, which can be low and governed by heuristics or high and governed by deliberation. Both systems are activated, and reciprocally interact, during the process, and the interesting question is which one dominates the other.

The two approaches to communication that are more widely accepted today, the Heuristic Model (Chaiken (1980)) and the Elaboration Likelihood Model (Petty and Cacioppo (1981)),^18^ both make reference to the dual model, and distinguish between systematic (or central route) processing and heuristic (or peripheral route) processing.

“Systematic processing implies that people have formed or updated their attitudes by actively attending to and cognitively elaborating persuasive argumentation. In contrast, heuristic processing implies that people have formed or changed their attitudes by invoking heuristics”

“These dual-process theories regard systematic processing as more effortful and capacity limited than heuristic processing. They therefore assume that heuristic processing predominates when motivation or capacity for effortful processing is low; for example, when the issue or one’s judgment is inconsequential [..] or when time does not permit extensive information processing.” Chaiken and Maheswaran (1994), 460

It is easy to recognize that the impact of advertising is particularly significant - as all of us experience in our daily lives - as the recipient of the persuasive message offers a cognitively low-level reaction, displaying a predominance of heuristic processes over rational ones.

This idea emerges clearly from Schumpeter’s picture of political decision making:

‘The only point that matters here is that, Human Nature in Politics being what is, [leaders] are able to fashion and, within very wide limits, even to create the will of the people. What we are confronted with in the analysis is not a genuine but a manufactured will. And often this artefact is all that in reality corresponds to the *volonté générale* of the classical doctrine. So far as this is so, the will of the people is the product and not the motive power of the political process.

*The ways in which issues and the popular will on any issue are being manufactured is exactly analogous to the ways of commercial advertising*. We find the same attempts to contact the subconscious. We find the same technique of creating favorable and unfavorable associations which are the more effective the less rational they are. We find the same evasions and reticences and the same trick of producing opinion by reiterated assertion that is successful precisely to the extent to which it avoids rational argument and the danger of awakening the critical faculties of the people. Schumpeter (2003), 263, italics added

Schumpeter emphasizes the negative effects of persuasion when it takes place in a hidden way without allowing rational control by citizens, but also offers remedies for the most negative effects of hidden persuasion and advertising. He suggests that the main remedy is the intelligent and critical use of the internal mechanisms of democracy. This means that a reciprocal rivalry is vital to induce political leaders to offer credible programs to voters, by using many different forms of persuasion.

To identify better these forms of persuasion, it is important to distinguish clearly the different frames in which persuasion takes place, and the consequent impact it may have on individuals and institutions:

Persuasion can happen:

- in a context of reciprocal trust and in an explicit setting, as in the teacher-scholar relation, in which critical debate explicitly assumes an essential role^19^;
- in a context in which one part seeks explicitly to modify the beliefs or choices of the other part through discussion, as in the lawyer-judge relation.
  This relation implies persuasion: since medieval times, debates in lawsuits have been an exercise in persuasion, with the use of rhetoric. The judge in a debate must decide which side’s position is more plausible in light of the arguments given, and the lawyers can use persuasive reasoning, weaving a discussion that can entail the deliberate use of fallacies. 20
  Persuasion in this case is based on a sophistic use of rationality through the art of rhetoric, at times with an attempt to induce the opponent into biased reasoning; but in the above case, persuasion is conducted under fair conditions because both parts are aware of possible hidden *sophisms*.
- in a context in which one part aims implicitly to modify the beliefs or the choice of the other part, through a sort of communication largely centered on emotional messages (which is the typical style of modern advertising)^21^;
- in a context in which one part aims implicitly to modify the beliefs or the choice of the other part by modifying his decisional context through so called *nudging*.^22^

All the above modes of persuasion imply a permanent *dualism* between *autonomous* and *induced* individual will. It is clear that persuasion is compatible with autonomous will only on condition that both parts in the relation are aware of the possible unintended and uncontrolled effects of persuasion, as in the first two instances; in these cases, individual will develops autonomously, while in the other cases, it is won over by a form of persuasion that weakens rationality. Thus, as the dual model suggests, a way to foster the level of people’s cognitive skills is to create conditions in which the interaction between intuitive and logical processes of thinking is strongly activated. This may be done by an individual in isolation - with a slow and strenuous process, as we have noted before - but it will be much more fruitful if stimulated and reinforced through critical discussion with other persons.

Unfortunately, this is a very difficult and slow process, and Schumpeter considers it highly unlikely to happen.

“[…..] Without the initiative that comes from immediate responsibility, ignorance will persist in the face of masses of information however complete and correct. It persists even in the face of the meritorious efforts that are being made to go beyond presenting information and to teach the use of it by means of lectures, classes, discussion groups. *Results are not zero. But they are small*. People cannot be carried up the ladder”. Schumpeter (2003), 262

The recent research on debiasing has fully confirmed that public information campaigns designed to dispel erroneous beliefs and to replace them with more accurate information, largely fail to achieve their goal. 23

“One piece of this puzzle is that increased effort will only improve performance when people already possess strategies that are appropriate for the task at hand; in the absence of such strategies, they will just do the wrong thing with *more gusto*”. Schwarz et al. (2004), 128.

Here the central role of leadership emerges: proposing new strategies, and using persuasive methods to achieve consensus. The problem is to what extent persuasion can rest on rationality. Given the existing average gap of competence, according to Schumpeter, most voters cannot be persuaded through purely rational arguments, and thus advertising is a necessary instrument for mass communication.

“The psycho-technics of party management and party advertising, slogans and marching tunes, are not accessories. They are of the essence of politics. So is the political boss.” Schumpeter (2003), 283

There is an inherent ambiguity within the system of communication between leaders and citizens. The political communication systems, based on the psycho-technics of persuasive communication, facilitate a misuse, which permits the conveyance of biased information

“Since the first thing man will do for his ideal or interest is to lie, we shall expect, and as a matter of fact we find, that effective information is almost always adulterated or selective and that effective reasoning in politics consists mainly in trying to exalt certain propositions into axioms and to put others out of court”. Schumpeter (2003), 264

Thus communication and persuasion, which are essential for democracy, also have an ambiguous and potentially disruptive role, which can be contrasted only if the slow process through which citizens increase participation is rooted in the growth of conscious rationality.

## Revisiting Schumpeter’s vision of democracy

The Schumpeterian picture of democracy rests on the imperfect competence of citizens and the imperfect institutional mechanisms that put them in relation with the leaders, i.e., the ambiguous role of communication and advertising. He does not bestow upon democracy a salvific property: unlike the classical approach, he warns that the core problem of democracy is how to achieve rational collective decisions despite the competence gap of the citizens.

Acknowledging that a part of thought processes are not under our deliberate control, Schumpeter warns that achieving clear and free will is not an easy task: it requires citizens in politics, as well as consumers and traders in economics and in finance, an active and permanent effort that can be either fostered or hindered by the institutions. Persuasion, as we have seen, can in fact have a very important and “positive” role in allowing citizens to clarify their opinions and preferences. It can allow leaders to balance divergent positions amongst citizens and achieve a common will. However, it can also have negative effects, as it may turn into an intrusion and disguised alteration of citizens’ preferences and wills.

The strong emphasis that Schumpeter places on the latter, perverse, effect is unmistakable; a kind of pessimism only apparently similar to Nietzschean positions.^24^ Unlike Nietzsche, Schumpeter recognizes the permanent dynamics between autonomous and induced will, and depicts democracy as an institution that allows people to achieve political decisions without necessarily having a high degree of awareness and competence. This is, in my view, the strength of the Schumpeterian theory of democracy: in fact, he makes it clear that a defining property of democracy is that it is an institution that may be working even if the degree of awareness, competence and analytical effort of its citizens is low. Thus the main problem that Schumpeter has to solve in sketching the fundamentals of democracy is conceiving institutions that allow rational, or at least reasonable, public decisions *despite* the substantial limits of individuals’ rationality.

The above conclusions on the conditions under which persuasion does *not* lead to a manufactured will suggest that fostering critical debate among citizens and political groups through appropriate internal institutions and regulations is vital for democracy; democracy must enable citizens to make their political assessments and choices in a more rational manner. If political institutions allow leaders to emerge through pacific competition, through debates and discussions, voters can achieve a reasonably clear opinion about the issues in discussion. This is the function of democracy for Schumpeter: ensuring a process of political competition that works properly, so that leaders enjoying broad consensus can assume power.

“The democratic method is that institutional arrangement for arriving at political decisions in which individuals acquire the power to decide by means of a competitive struggle for the people’s vote.” Schumpeter (2003), 269

Naturally, an opposite process of *adverse selection in politics* is permanently at work.^25^ There is a possibility that the world of politics deceives citizens, that voters are systematically sold ‘lemons’ by leaders who make vague promises, who make massive use of covert persuasion techniques, who substitute advertising for political debate: this generates a process of adverse selection in politics, which may induce citizens to withdraw from the electoral market. In this case, participation declines to the point when democracy as an institution may collapse, which happens if adverse selection covers all different parties in the game (i.e., if competition becomes collusion for political rent). Schumpeter clearly warns about this risk for democracy in conditions of low rational critical debate.^26^

“And there is truth in Jefferson’s dictum that in the end the people are wiser than any single individual can be, or in Lincoln’s about the impossibility of “fooling all the people all the time.” But both dicta stress the long-run aspect in a highly significant way. It is no doubt possible to argue that given time the collective psyche will evolve opinions that not infrequently strike us as highly reasonable and even shrewd. History however consists of a succession of short-run situations that may alter the course of events for good. If all the people can in the short run be “fooled” step by step into something they do not really want, and if this is not an exceptional case which we could afford to neglect, then no amount of retrospective common sense will alter the fact that in reality they neither raise nor decide issues but that the issues that shape their fate are normally raised and decided for them. More than anyone else the lover of democracy has every reason to accept this fact and to clear his creed from the aspersion that it rests upon make-believe.” Schumpeter (2003), 264 27

The problem is, therefore, to what extent a manipulated choice is intolerable to democracy, and the possible remedies. Schumpeter claims that economic competition shows characteristics similar to those typifying political competition in modern societies: on the one hand, democracy is depicted as a system ensuring that the struggle for power takes the peaceful form of political election, and as a method for the selection of leaders. 28 On the other, the market is conceived as the system through which the struggle for profit is led “in a pacific way”, based on competitive processes that elicit innovators/leaders.

On these premises, Schumpeter’s view allows us to consider both systems - market and democracy - in a comprehensive way: he implicitly claims that exactly the same social mechanisms and human micro-properties work in either system, the main differences lying in the degree of conscious rationality that individuals exhibit and the extension and intensity of competition in the two different domains.

In conclusion, with its conceptual unification of economic and political behavior, Schumpeter’s analysis warns that virtuous selection and adverse selection are ever-present in the market and in politics. Democracy is therefore a vulnerable institution. A key element we have identified for understanding the threshold between the stability and decline of democratic institutions is the competence gap between citizens and political leaders: only if the competence gap is balanced by political leaders striving for transparency and clarity can democracy safely run. But the behavior of political leaders is not virtuous by nature or by law, and only a continuous working of competitive pressures upon and among the leaders can foster the rational elements in political debate and the growth in the long run of the self-determination of the will of citizens. 29

This picture of democracy testifies to the extreme modernity of Schumpeterian approach, which sheds light on the essential and ambiguous role of communication and persuasion, an element that nowadays influences the quality of democracy in a more and more sophisticated way.

## Appendix : Wason selection task

Briefly, the selection task works as follows:

Subjects are shown four cards, each with a letter on one side and a number on the other. The exposed card faces are A, B, 2 and 3. Subjects are asked which cards should be turned over to test the following conditional rule: If there is an A on one side there is a 2 on the other.

Few subjects give the right answers: A and 3. In fact, if a player turns A and on the opposite side there is 3, then the rule is falsified; analogously the rule is falsified if he turns the 3 and on the opposite side there is an A. Thus the right thing to do is to turn the cards that could show a *violation* of the rule. But the majority of people pay attention to the cards that must be turned in order *verify* the rule, which are A and 2.

However, most subjects give the right answer when the logically equivalent problem is put in a form that induces individuals to check the cards that falsify the rule: this is made by creating a context in which the Wason task is interpreted as a search for violations of a rule. Cox and Griggs (1982) carried out an experiment with a police officer observing people drinking in a bar who must detect which customers are violating the rule that teenagers cannot drink alcoholic beverages. Therefore the “cheating detection rule”, is elicited by the form in which the problem is presented and leads them (inadvertently) to select the right cards for the solution of the problem. Griggs and Cox reported around 75 % successful answers.

Such a result has been further generalized by Gigerenzer and Hug. The “perspective effect” that they introduce presents the Wason Selection Task in the form of an employer-employee contractual relation, and explores the effects of changing the role of the subjects involved in the contract. Gigerenzer’s thesis is that a “cheating detection mechanism” guides reasoning in the following type of selection task: if the conditional statement is coded as a social contract, and the subject is cued into the perspective of one party in the contract, and attention is directed to information that can reveal that one is being cheated. This thesis has been proven by comparing two different versions of the selection task, changing the subject that can be cheated in the contractual relation. (Gigerenzer and Hug 1992). This shows that the context in which the problem is framed may be an implicit guidance or impediment to solving a problem, without the subject’s awareness.

## Footnotes

1 The paper was discovered by Professor Loring Allen of the University of Missouri in St. Louis among Schumpeter’s papers in the Harvard University archives. For a wide analysis of the various aspects of rationality that Schumpeter discusses in the paper, see Festré and Garrouste (2008). In what follows I will focus on one specific aspect of his views on the topic, the meaning of “conscious rationality”, which has been largely neglected by commentators thus far.

2 Schumpeter (1947) 585

3 See, in particular, Simon (1985) who explicitly attributes “procedural rationality” to political decision making, characterized by inconsistencies or incompleteness. A similar view can be found in Arena R. Danger-Hagnauer C. (Eds) (2002), 59

4 This means that not all potentially available strategies can be discovered with the current computing power.

5 “As genetics needs its model organisms, its Drosophila and Neurospora, so psychology needs standard task environments around which knowledge and understanding can cumulate. Chess has proved to be an excellent model environment for this purpose”. (Simon and Chase 1973, 394)

6 See Heiner (1983), 562

7 These requirements could be translated in terms of the difficulty of computing a problem. An interesting attempt to give a formal representation of the problem of non-computability in economics is due to Velupillai (2000).

8 Dosi and Egidi (1991); see also Dequech (2001)

9 See the early experiments by Schneider, W. and Shiffrin, R. (1977)

10 “During the second half of the last century, the idea of the human personality that is a homogeneous unit and the idea of a definite will that is the prime mover of action have been steadily fading—even before the times of Théodule Ribot and of Sigmund Freud”. (Schumpeter (2003), 256)

11 “The theory argues that affective somatic states associated with prior decision outcomes are used to guide future decisions. For example, when a choice followed by a bad outcome occurs, an affective reaction becomes associated with that choice. Once the affective reaction is sufficiently well-established, the reaction occurs before a choice is made. Anticipation of a bad outcome before the bad choice is made prevents the bad choice and leads, instead, to a better choice.” Hinson, Jameson, and Whitney (2002), 341. See also Damasio, Tranel, & Damasio (1991)

12 Wason (1966)

13 Evans et al. (1983)

14 Kahneman (2002), 453

15 Citizens – or a majority of them - are supposed to exert a low level of *conscious rationality* when debating politics. This means that they have a high competence gap in this domain, which limits their analytical skills: they cannot use their problem-solving attitudes because of their ignorance in politics, not because they are irrational. For this reason, while I share many aspects of the interpretation that Manfred Prisching (1995) gives to Schumpeter’s Theory of Democracy, I would not define Schumpeter as the founder of the “irrational choice” theory. Simon (1985) illustrates very convincingly this point especially in the paragraph “Bounded Rationality is not Irrationality”, 297 and 301. See also Dequech (2001) 216.

16 This picture has been fully confirmed by experimentation in psychology, and important literature makes clear the reasons for and conditions of this failure. See also the next section of the present article.

17 One of the most noteworthy accounts is given by the Ryan and Deci Self-Determination Theory. They distinguish between different types of motivation based on the different reasons or goals that give rise to an action. “The most basic distinction is between *intrinsic motivation*, which refers to doing something because it is inherently interesting or enjoyable, and *extrinsic* motivation, which refers to doing something because it leads to a separable outcome”. Ryan and Deci (2000) 54

18 Petty and Cacioppo suggest that “there are’central’ and ‘peripheral’ routes to persuasion, with the’central route’ representing the processes involved when elaboration likelihood is high and the’peripheral route’ typifying the processes operative when elaboration likelihood is low”.

19 See also Greenwald (1968)

20 Going under the name of “fallacies”, deviations from logically correct reasoning have been widely analyzed since the XII Century; with the translation into Latin of *De Sophisticis Elenchis* (the last part of Aristotle’s *Organon*) many scholars have attempted to detect, describe, classify and analyze fallacious arguments. According to Hamblin: “A fallacious argument, as almost every account from Aristotle onwards tells you, is one that seems to be valid but is not so…. Greek has no precise synonym for ‘fallacy’, and the word so translated is generally *sophisma*; ….. That Aristotle is writing about *deliberate* sophistry, and not about mere errors or mistakes, is made clear quite early” Hamblin (1970), 12

21 On the connection between political leaders, discursive strategies and advertising techniques, see Landowski (1989) who focuses on the different meaning effects through which political leaders build and renegotiate their identity and public profile using different kind of texts.

22 In their book, *Nudge*, Thaler and Sunstein (2008) suggest several ways in which government agencies and private organizations might “nudge” individuals toward actions that are better for them “*as judged by themselves*”. They call this policy “libertarian paternalism”, claiming that it does not threaten liberty, because it does not hinder individuals from choosing what they prefer. Whatever specification and mitigation could be added to this approach, it is difficult to avoid the doubt that nudge factors are interferences with rational choice, rather than rational determinants of choice, because of the hidden nature of the way they function.

23 For a review, see Rice & Atkin (2013).

24 “The strongest knowledge — that of the total unfreedom of the human will — is nonetheless the poorest in successes, for it always has the strongest opponent: human vanity. “Nietzsche (2006) 275

25 “Political representation is an outstanding example of the principal-agent relation. This means that the link between individual utility functions and social action is tenuous, though by no means completely absent. Representatives are no more a random sample of their constituents than physicians are of their patients”. Arrow (1969) 13

26 More on this point in Egidi (1996)

27 This view depicts a very interesting process: advertising, communicating and persuading may distort the conditions of choice in a way that gives rise to “hysteresis”, where hysteresis is a typical “history dependent” characteristic of the magnetization of ferromagnetic materials. So far this type of process has relevance in economics only in some of the papers of Blanchard and Summers (1986)

28 See Schumpeter (2003), Section I of Chapter XII. Unlike Adam Smith’s idea of attributing to empathy the foundations of social order, he implicitly suggest that competition socially fosters human fairness and provides the foundations for democracy. For a wide discussion on this point, see McCann (1999).

29 Therefore, I consider misleading the attribution of an “elitist” character to the Schumpeterian view of democracy. This attribution comes from a static interpretation of his dynamic/evolutionary description of the working of the internal mechanisms of democracy. These mechanisms, insofar are based on a complex system of communication and interaction between citizens and representatives, reveal the ambiguous character and the fragility of democratic processes of decision. See Medding (1969) for a broad discussion on the elitist views.
